# Avascular necrosis following closed reduction for treatment of developmental dysplasia of the hip: a systematic review

**DOI:** 10.1007/s11832-016-0776-y

**Published:** 2016-11-03

**Authors:** Catharine S. Bradley, Daniel C. Perry, John H. Wedge, M. L. Murnaghan, Simon P. Kelley

**Affiliations:** 1 Division of Orthopaedic Surgery, The Hospital for Sick Children, 555 University Avenue, Toronto, ON M5G 1X8 Canada; 2 Institute of Translational Medicine, University of Liverpool, Eaton Road, Liverpool, L12 2AP UK

**Keywords:** Developmental dysplasia of the hip, Congenital hip dislocation, Closed reduction, Avascular necrosis, Systematic review

## Abstract

**Background:**

Avascular necrosis (AVN) is a significant and potentially devastating complication following the treatment of developmental dysplasia of the hip (DDH). The reported rate of AVN following closed reduction for DDH ranges from 4 to 60%, and the resultant influence on hip development remains unclear.

**Purpose:**

A systematic review of the literature was undertaken to evaluate the frequency of AVN after more than 5 years of follow-up in children that underwent closed reduction at younger than 2-years of age for DDH.

**Methods:**

The search strategy was formulated with key-concepts and keywords identified using the patient problem, intervention, comparison and outcome process. Searches were undertaken using Pubmed, Scopus and Web of Science up to and including May, 2016 to identify potential studies.

**Results:**

A total of seven papers met the a priori inclusion and exclusion criteria of this review. The overall rate of significant AVN in 441 patients (538 hips) was 10% at a mean length of follow-up of 7.6 years (5–18.8) following closed reduction. This finding can be used to inform the feasibility of future intervention studies, and act as a baseline for which surgeons to compare their results to a ‘standard’.

**Electronic supplementary material:**

The online version of this article (doi:10.1007/s11832-016-0776-y) contains supplementary material, which is available to authorized users.

## Introduction

Closed reduction and spica casting is one of the most commonly performed procedures for the treatment of developmentally dislocated hips (DDH). Whilst the procedure may be considered ‘minimally invasive’ and commonplace, it is not without complications. The primary complication of closed reduction is avascular necrosis (AVN) of the femoral head that occurs due to diminished blood supply to the femoral epiphysis that can cause devastating clinical outcomes [1–7]. The aetiology of the AVN in closed reduction is thought to be a positional vascular occlusion in the spica cast, though the position-at-risk varies by child, and determination of this position cannot yet be routinely individualized. The ‘optimal position in spica cast’ therefore relies on a best-fit approach; using the safe-zone of Ramsey [8].

Reported frequency estimates of AVN following closed reduction vary significantly between 4 and 60% [1–7]. There is therefore a significant discrepancy in the literature, with difficulty in interpreting the expected rate of AVN. Whilst this variation may be a consequence of natural variation due to the relatively small case numbers, it may similarly be due to systemic differences such as case selection, or surgical technique (i.e. cast position, duration of treatment, tendon release). A clear understanding of the expected AVN rate would empower surgeons to audit their own practice against a ‘standard’, would provide feasibility data for the measure of effect size in future intervention studies, and may help to elicit if there are particular aspects of the surgical intervention that may particularly heighten the risk of AVN.

The purpose of this study was to systematically evaluate the literature of children having underwent closed reduction under 2-years of age for DDH, to determine the frequency of AVN after more than 5-years of follow-up.

## Methods

### Search strategy and criteria

The search was conducted within Pubmed, Scopus and Web of Science up to and including May, 2016. The search strategy was formulated with key-concepts and keywords identified using the patient problem, intervention, comparison and outcome (PICO) process [9]. This identified essential search-terms, which were exploded ensuring the inclusion of relevant synonyms, alternative spellings and related terms [10]. Individual search terms were combined using Boolean technique to further refine the process. A medical librarian was instrumental in helping to design the search strategy.

Initial search keywords were broad and exploded terms, to ensure full use of MeSH (Medical Subject Headings) terms for maximum sensitivity. More specific terms and limitations were subsequently introduced and combined to refine the search [11] (Appendix 1).

For inclusion in the review, articles needed to be reports of clinical studies of DDH treated by closed reduction in human patients and a minimum series of ten hips. A minimum of ten hips was selected to minimize the effect of small sample bias in the overall analysis. The minimum follow-up period was 5 years, therefore this must either have been a feature of the study design, or the study must have published sufficient data such that individual cases with over 5 years of follow-up could be elicited. Shorter follow-up periods were not considered, as later-onset AVN, and more specifically type 2 AVN, that may not evident until several years after surgery, could not be excluded. Additionally, all studies had to report on AVN using a recognized and previously published classification of AVN. Exclusion criteria were made if studies reported on closed reductions performed in children over 2 years of age (unless individual patient data was available to include such patients), and studies of children with teratologic hip dislocations (i.e. fixed hip dislocations at birth that are associated with congenital anomalies, other syndromes or neuromuscular disease) as closed reduction is typically not recommended in any of these subgroups. In addition, the search was limited to studies in English.

Titles and abstracts were independently screened against the inclusion criteria by two investigators with prior experience of conducting systematic reviews (XX and YY). If either investigator deemed that the title and abstract fulfilled the inclusion criteria, then the full paper was obtained. The same reviewers then screened the full papers against the inclusion and exclusion criteria and the reason that any paper failed to fulfill these criteria was noted. The senior author (ZZ) resolved any disagreement between the two reviewers. The reference lists of the included papers and of any identified review articles were also assessed for further relevant studies.

Data was extracted from those studies included independently by the two investigators using a Genaidy Critical Appraisal Instrument [12]. The detailed data from these forms were entered into an excel spreadsheet to allow for an assessment of heterogeneity and quality between studies.

### Outcome reporting

Common AVN classifications systems have previously been combined in the following manner [13]: Type 1 AVN is identical in the Kalamchi and MacEwen, and Bucholz and Ogden classifications, but does not result in long term disease and has therefore not been considered clinically significant AVN. Type 2 AVN is also identical in both classifications and is thus considered together. Types 3 and 4 in each classification were recorded as clinically significant, and combined in a type 3 group. Salter classification—in instances where the location of physeal damage is reported, ‘no physeal damage’ is considered type 1, ‘lateral physeal damage’ is considered type 2 and ‘central physeal damage’ type 3 AVN. For the purposes of this study, only types 2–4 AVN are considered clinically significant and type 1 AVN is not included in the analysis.

### Statistical analysis

The overall frequency of and type of AVN (types 2–4) related to length of follow-up were evaluated across all included studies. Mean age at closed reduction in months, mean length of follow-up in years, percentages for gender and AVN classification system used were identified for each study.

## Results

**Fig. 1:**
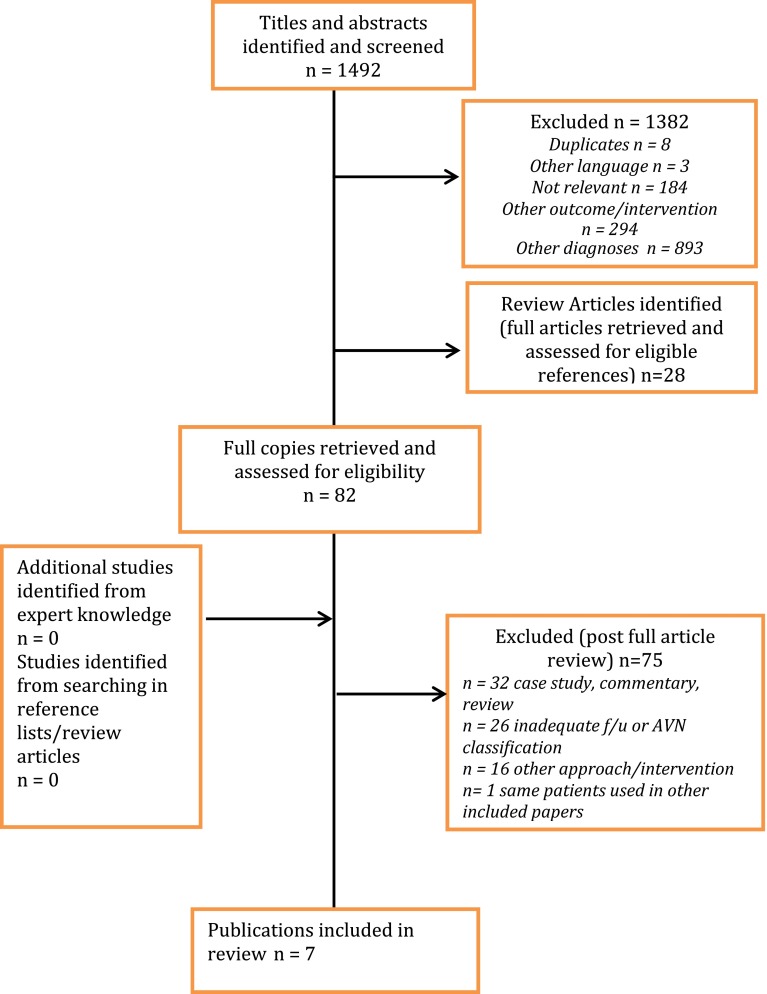
Literature search results

The search identified 1492 possible titles and abstracts (Fig. 1). Initial review of these excluded 1382 articles and identified 28 review articles to further assess the included references. This resulted in the retrieval of 82 full papers for confirmation of eligibility. Searching references lists and conferring with experts did not add any further articles. Review of the full papers excluded an additional 72 articles leaving 7 articles (538 hips) for analysis. Four of these articles met inclusion criteria for all patients in their respective studies [14–17] and the remaining three provided sufficient individual patient data to be retrieved and included in the analysis [18–20].

### Data extraction

All seven studies were reports of case series and all but two [14, 19] were formed retrospectively. All studies reported outcomes of closed reduction for DDH including AVN. Exclusion criteria for the studies included teratologic hips and prior attempts at closed reduction. Most were conducted at a single institution, one at two medical centers [15] and one at four centers [16]. Summary details of each of the studies are provided in Table 1.

**Table 1:** Summary of included studies

References	Patients (*n*)	Affected hips (*n*)	Female (%)	Mean age at reduction (months)	Mean follow-up (years)	AVN classification	Rate of significant AVN (%)
Bicimoglu et al. [16]	143	185	88.8	11.6 (3–18)	7.5 (5–13)	K&M	5.4 (10/185)
Carney et al. [18]	32	35	77.1	8.2 (1–21)	8.75 (5.3–13.6)	B&O	37.1 (13/35)
Cooke et al. [15]	42	48	92.9	10.2 (2–20.6)	11.1 (5–18.8)	K&M	2.1 (1/48)
Danielsson [19]	65	67	89.4	10 (2–20)	11.7 (6.2–18.2)	Salter K&M	6 (4/67)
Forlin et al. [20]	28	33	89.3	13 (3–21)	7.3 (5.3–12.6)	K&M	6 (2/33)
Khoshhal et al. [14]	85	124	69.4	7.3 (3–14)	“Minimum 5 years”—no further details given.	K&M	10.5 (13/124)
Pospischill et al. [17]	46	46	83.3	4 (1.2–10.4)	6.3 (5.2–7.2)	B&O	19.6 (9/46)

AVN classification; *K&M* Kalamchi and MacEwen, *B&O* Bucholz and Ogden

### Patient characteristics

Of the 441 patients (538 hips), 86.5% were female and 76.2% were unilateral in presentation. The mean age at time of closed reduction was 9.6 months (range 1–21) with a mean follow-up of 7.6 years (range 5–18.8) after closed reduction.

### Interventions

Previous treatment with a Pavlik harness was reported in three of the studies [15, 17, 18] and pre-operative traction was used at varying rates in all but one study [16]. The closed reductions were performed either by a staff surgeon or under their supervision. Soft tissue releases were not routinely performed in all studies, but when completed, adductor longus was the only one noted. Reported time in spica ranged from 6 weeks [14] to 6 months [19]. Position of immobilization also ranged from a combined 100° of flexion, 20° of abduction [15] to combined flexion of 100°–110°, 40°–60° of abduction and no internal rotation [17]. None reported using abduction greater than 60°.

### Overall rate of AVN

Significant AVN occurred in 52 of the 538 hips included (441 patients), which equates to an AVN rate of 10% for hips with a minimum of 5-years of follow-up [mean duration of follow-up of 7.7 years (range 5–18.8)].

### Time

**Fig. 2:**
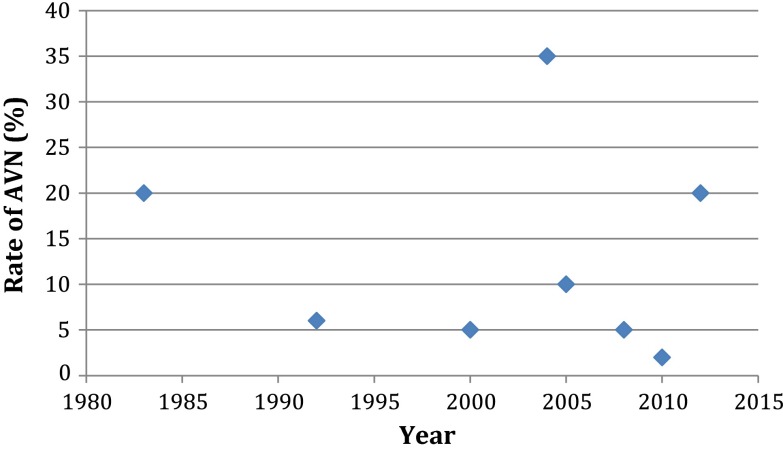
Scatter plot illustrating the relationship between year of publication and the rate of types 2–4 AVN

There was no apparent temporal relationship between AVN rate, and year of publication (Fig. 2). This suggests that there was no significant change in practice over the inclusion period, which significantly influenced AVN rates.

### Size of study

**Fig. 3:**
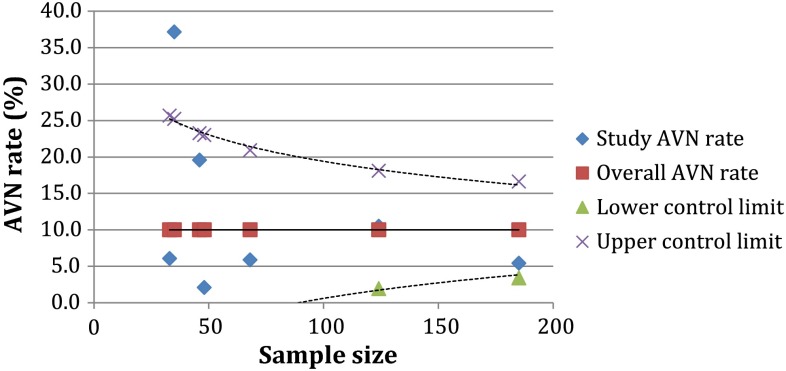
Funnel plot to illustrate the rate of types 2–4 AVN, according to study sample size

There was a marked change in the AVN rate seen depending on the size of the study; which is largely a feature of common cause variation, with larger studies having more certainty, and therefore narrower confidence intervals. This is demonstrated using a funnel plot in Fig. 3. The funnel plot indicates that the target line for the predicted ‘normal’ rate of AVN (types 2–4) following closed reduction is 10%. The control limits, drawn using three standard deviations, demonstrate that much of the variation within the published literature is explained by common cause variation, as the results lie within the control limits. One study fell significantly outside the control limits [18].

## Discussion

This is the only review that has synthesized long-term AVN outcomes following closed reduction for Developmental Dysplasia of the Hip (DDH). This study has demonstrated that the mean rate of AVN within the literature is 10% after 5-years follow-up after closed reduction, amongst children treated before 2 years old. This result offers a ‘target’ against which clinicians may compare their results and offers a summary measure for use when powering intervention studies.

The wide variations in the published results of AVN, is likely to be largely a consequence of common cause (natural) variation [21]. Common cause variation cannot be eliminated, and reflects the confidence or certainty within a sample. As the sample size is increased, the certainty increases, and the confidence interval narrows; hence a funnel is formed. Data points occurring outside the funnel are special cause variations, which indicate that an extrinsic cause is influencing the outcomes seen. Special cause variation may be due to differences in technique, surgeon factors or systematic differences in the way that a study is run. The result of one of the papers within this review fell significantly outside the control limits, indicating ‘special cause variation’ [18]. This may therefore indicate that there was a fundamental difference in some part of intervention offered to these participants within this study, or could be a consequence of a bias in the study design (i.e. cases with AVN may have been more readily identified and recruited owing to more frequent follow-up visits). Such special cause variations require further investigation in order to identify and act upon the special cause.

With increasing transparency within surgery, it is important that surgeons are able to audit their results against a gold standard by which they can benchmark. Likewise, it is also important that they understand the concept of natural variation, such that they are able to consider their results within the context of the number of procedures that they undertake; thereby ensuring that their benchmarking is appropriate. Internationally, arthroplasty surgeons are perhaps most used to this type of scrutiny, as the volumes by which they undertake procedures sufficiently narrows confidence intervals in order to readily identify outliers and act accordingly. Whilst this may be somewhat more difficult within paediatric orthopaedics, efforts to increase transparency and audit results (by individual or centre) should be encouraged.

There may be specific aspects of the intervention that may have a bearing on outcome that cannot be well ascertained given the nature of this review and the included studies. Other previous studies, not included within this review, have made observations, particularly regarding the importance of the presence of the ossific nucleus prior to closed reduction [22, 23], though it remains controversial [24]. In addition, there may be adaptions within the surgical technique, such as routine adductor tenotomy and duration and position of immobilization in spica casting that may minimize the complication of AVN. This suggestion warrants that well designed prospective analysis of potential predictive factors. In association with the International Hip Dysplasia Institute (IHDI), there is an ongoing multicentre nationwide trial within the UK that is seeking to address the timing of the intervention (i.e. immediate vs. delayed after the appearance of the ossific nucleus). The results of this, and other similar trials, may have profound effects for the way that we manage DDH [25].

A systematic review such as this, attempts to harmonize the results of a number of studies, by strictly defining inclusion/exclusion and intervention variables. Whilst this is useful to gain a summary measure, flaws within individual studies are difficult to overcome. These flaws are particularly apparent for retrospective case series, whereby the population was never clearly defined, case ascertainment was unclear, potential confounders were not recorded (i.e. position in spica) and the methodological clarity was limited. The review was also limited by the analysis of only literature written in English, and the paucity of studies that have reported long-term follow-up. Nevertheless, the study of rare outcomes is challenging in a prospective manner [26], owing to the costs and infrastructure required to identify and follow-up cases in a systematic manner, therefore a pragmatic approach must be considered.

This review has enabled individual surgeons/centres to benchmark themselves against the most robust studies of outcome within the literature and has set a summary measure against which future studies may compare outcomes.

## Electronic supplementary material

Below is the link to the electronic supplementary material.

Supplementary material 1 (DOCX 13 kb)
