# Supplementary material for: Avascular necrosis following closed reduction for treatment of developmental dysplasia of the hip: a systematic review
Source: J Child Orthop. 2016 Nov 3;10(6):627–32. doi: 10.1007/s11832-016-0776-y (PMC5145826; doi:10.1007/s11832-016-0776-y)
Supplement: Supplementary file 1 — Supplementary material 1 (DOCX 13 kb) [file 11832_2016_776_MOESM1_ESM.docx]

**Appendix 1: Database Search Terms**

(1) Dislocation

(2) Dysplasia

(3) Displasia

(4) 1 OR 2 OR 3

(5) “Closed Reduction” (including a proximity search on ‘closed reduction’; whereby ‘closed’ and ‘reduction’ must be separated by 3 words or less).

(6) Spica

(7) Surgical Casts

(7) 6 OR 7

(8) hip

(14) 4 AND 7 AND 8

**String (Pubmed)**

(spica OR surgical casts OR “closed reduction”) AND (dislocation OR dysplasia OR displasia) AND hip

**String (Ovid Web of Science)**

(spica OR surgical casts OR (closed w/3 reduction)) AND (dislocation OR dysplasia OR displasia) AND hip

**String (Scopus)**

(spica OR surgical casts OR "closed w/3 reduction") AND (dislocation OR dysplasia OR displasia) AND hip
